# ABCA1 Deletion Does Not Affect Aqueous Humor Outflow Function in Mice

**DOI:** 10.1155/2024/7195550

**Published:** 2024-07-17

**Authors:** Bo Qin, Chunchun Hu, Youjia Zhang, Yuhong Chen, Yuan Lei

**Affiliations:** ^1^ Department of Ophthalmology and Visual Science Eye and ENT Hospital Shanghai Medical College Fudan University, Shanghai, China; ^2^ Key Laboratory of Myopia Chinese Academy of Medical Sciences Fudan University, Shanghai, China; ^3^ Shanghai Key Laboratory of Visual Impairment and Restoration Fudan University, Shanghai, China

## Abstract

**Background:**

ATP binding cassette transporter A1 (ABCA1) is a candidate gene within a POAG susceptibility locus by GWAS analysis, and it is involved in IOP modulation via the Cav1/eNOS/NO signaling pathway. We aim to examine the phenotype of ABCA1 deletion in the ABCA1 gene knockout (Abca1^−/−^) mice.

**Methods:**

The anterior segments of Abca1^−/−^ eyes were imaged by slit-lamp microscopy and anterior segment OCT. IOPs were measured by rebound tonometry. By perfusing enucleated eyes at various pressures, the aqueous humor outflow facility was determined. The mRNA expressions of ABCA1, Cav1, and eNOS were measured by RT-qPCR. The protein expressions were analyzed by western blot and immunofluorescence staining.

**Results:**

There was no significant difference in the anterior segment morphology of Abca1^−/−^ mice. IOP and aqueous humor outflow facility did not change in Abca1^−/−^ mice compared with wild-type mice. mRNA and protein expressions of ABCA1 were significantly lower in the outflow tissue of Abca1^−/−^ eyes. The expressions of Cav1 and eNOS were both significantly upregulated in the outflow tissue of Abca1^−/−^ eyes.

**Conclusion:**

ABCA1 deletion does not affect IOP and aqueous humor outflow function but the Cav1/eNOS/NO pathway is changed in Abca1^−/−^ mice. The function of ABCA1 in aqueous humor outflow still requires further research.

## 1. Introduction

Glaucoma is an irreversible and progressive degenerative disease of the optic nerve, and it is the second blinding eye disease in the world [1, 2]. Primarily inherited as a complicated trait, primary open-angle glaucoma (POAG) is one of the most common types of glaucoma in the world. The main risk factor for POAG is elevated intraocular pressure (IOP) [3], which is characterized by the increased resistance to drainage of aqueous humor [4].

ATP binding cassette transporter A1 (ABCA1) is a member of a large superfamily of ABC transmembrane transporters that facilitates the efflux of cholesterol to lipid-free apolipoproteins A-I and E [5]. As a transmembrane protein that is widely expressed in many tissues, ABCA1 may have a variety of functions and contribute to the pathophysiology of numerous diseases [6, 7]. The reverse cholesterol transport (RCT) process is its most researched function [8]. Additionally, ABCA1 regulates the plasma membrane's levels of phospholipids and cholesterol and plays a role in particle production and cell signal transduction [9, 10].

We and other groups have found that ABCA1 is a potential gene for the POAG susceptibility locus [11–13]. Recent GWAS has identified genome-wide significant association at multiple SNPs near ABCA1 at 9q31.1 [11]. Gharahkhani et al. [12] and Hysi et al. [13] have shown that rs2472493, which is near ABCA1, has been linked to POAG and elevated IOP. Moreover, ABCA1 is involved in IOP modulation via the Cav1/eNOS/NO signaling pathway, which means a different mechanism from cholesterol efflux [14]. Also, in the retina of ABCA1 gene knockout (Abca1^−/−^) mice, cholesterol is accumulated and leads to a POAG-like phenotype [15]. The evidence suggests the pathogenesis of glaucoma, particularly in POAG, is significantly influenced by ABCA1 [11–15].

To further understand the function of ABCA1 in controlling IOP and aqueous humor drainage In Vivo, this study examined the phenotype of anterior segment morphology and aqueous humor outflow function of Abca1^−/−^ mice.

## 2. Materials and Methods

### 2.1. Animals

Our study complied with the ARVO Statement for the Use of Animals in Ophthalmic and Vision Research and was approved by the Eye and ENT Hospital Ethics Committee for Animal Experiments (IACUC-DWZX-2021-002). Abca1^−/−^ mice were purchased from the Model Animal Research Center (Nanjing University, Nanjing, China). These genetically modified mice's preceding generations have been described [16]. Wild-type (WT) DBA/2 mice were used as controls. Male mice aged 6–8 weeks were used in this study. Mice were bred and housed professionally in the animal house of the Eye and ENT hospital, Fudan University.

### 2.2. Slit-Lamp Microscopic Examination

Because of the next noncontact procedures, mice were hypnotized by intraperitoneal injections of 10 mL/kg of 4% chloral hydrate before examination. The slit-lamp microscopic examination (KSL-H3, Keeler, Windsor, UK) required two researchers. One ensured the position of the anesthetized mouse and the other observed and photographed the cornea, iris, conjunctiva, and anterior chamber of mice.

### 2.3. Anterior Segment Optical Coherence Tomography (AS-OCT)

Mice were examined by AS-OCT (Optovue, Fremont, CA) after slit-lamp microscopic examination under hypnotized status. We measured the central anterior chamber depth from the images recorded. After examination, the mice were placed on the thermal blanket until awakening from hypnosis.

### 2.4. IOP Measurement

By using rebound tonometry (TonoLab; ICare, Espoo, Finland), we measured the IOPs of live mice. IOPs were measured every day between 9:00 and 11:00 AM without anesthesia. For approximately 2 weeks before the experiments, the mice were acclimatized for IOP measurements. The researcher used one hand to gently keep the mouse's body still, and IOPs were measured by the other hand. The average of the three measurements of IOP was used as the IOP measurement for each eye.

### 2.5. Mouse Eye Perfusion

By perfusing enucleated mouse eyes, the aqueous humor outflow facility was determined. Our lab developed the experimental setup, which was extensively detailed elsewhere [17]. Briefly, a 33-gauge bevelled tip needle (stainless steel tip material; Nanofil; World Precision Instruments, Stevenage, UK) was used to cannulate the eye. The needle tip was placed in the anterior chamber. The eye was preperfused for 10 minutes under the pressure of 10 mmHg. Then the eye was repeatedly perfused for 15 minutes at four different pressures (6, 9, 11, and 16 mmHg). The measurement was performed twice for each pressure. At equilibrium, we assume that the total inflow rate and total outflow rate are equal. Goldmann's equation was used to compute the conventional outflow facility (*C*_con_): *F* = (IOP − EVP) *C*_con_ + *F*_*u*_, where IOP is the perfusion pressure, episcleral venous pressure (EVP) equals zero due to the enucleated eyes, *C*_con_ is the conventional outflow facility, and *F*_*u*_ is the unconventional outflow rate. On a flow rate-pressure response graph, a regression was fitted, and its slope was *C*_con_.

### 2.6. Western Blot

Mice were killed by neck dislocation, and within 5 minutes of death, the eyes were enucleated. Outflow tissue was dissected under a microscope. Trabecular meshwork (TM), Schlemm's canal, and perhaps some iris root were present in the dissected tissue. RIPA lysate (Beyotime, Shanghai, China) was used for preparing the outflow tissue samples. The Bradford method was used to estimate the protein concentration. By 12–20% gradient sodium dodecylsulfate polyacrylamide gel electrophoresis, equal amounts of protein (20 *μ*g protein/lane for mouse outflow tissue each) were separated. Electrophoresis was used to transfer the isolated proteins to PVDF membranes. The membranes were blocked using 5% nonfat dry milk in TBST (Sigma-Aldrich) at room temperature for 1 hour. Then, membranes were incubated using primary antibodies that specifically recognized ABCA1 (1 : 1000; Abcam, ab66217), eNOS (1 : 1000; Abcam, ab199956), Cav1 (1 : 1000; Cell Signaling Technology, #3238), or *β*-actin (1 : 3000; Cell Signaling Technology, 3700). *β*-actin was used as a loading control. Then blots were incubated with the secondary antibodies for 90 minutes. The blots were visualized by chemiluminescence.

### 2.7. RT-qPCR

The total RNA of outflow tissue was extracted using Tissue RNA Purification Kit (EZB, RN5). And mRNA was reverse transcribed using RT Mix Kit with gDNA clean for qPCR (ACCURATE BIOLOGY, AG11728). Next, quantitative real-time PCR was performed using SYBR Green Premix Pro Taq HS Kit (ACCURATE BIOLOGY, AG11701). PCR procedure was as follows: step 1, 30 sec was at 95°C; step 2, 40 cycles, done for 5 sec at 95°C, 30 sec at 60°C and collected fluorescent signals (Bio-Rad). The reference gene was GAPDH. The mRNA's relative expression levels were analyzed using the 2^−ΔΔCq^ method.

### 2.8. H&E Staining

Ocular sections were stained with hematoxylin solution for 5 min and then rinsed in distilled water. After being dipped in 1% acid ethanol and rinsed in distilled water, the sections were stained with eosin solution for 5 min, then dehydrated with graded alcohol, and cleared in xylene. An inverted confocal microscope (Leica) was used for imaging.

### 2.9. Immunofluorescence Staining

With 5% bovine serum albumin (Beyotime) and 0.1% Triton X-100 (Sigma-Aldrich), the ocular sections were blocked and made permeable for two hours at room temperature. The sections were treated with anti-Cav1 (1 : 50; Abcam, ab32577) or anti-eNOS (1 : 50; Abcam, ab300071) antibodies at 4°C overnight. They were then washed three times in phosphate buffer saline (PBS) and then incubated for two hours at room temperature with Alexa Fluor-488 or Alexa Fluor-555 goat anti-rabbit secondary antibodies (1 : 500; Invitrogen). Nuclei were visualized by DAPI (Sigma-Aldrich) counterstaining. All primary and secondary antibodies, as well as DAPI, were diluted in PBS containing 0.1% Triton X-100 and 1% bovine serum albumin. An inverted confocal microscope (Leica) was used for imaging. Each group had at least three imaging fields examined.

### 2.10. Statistical Analysis

Data normality was tested before comparison. The Student's *t*-test or ANOVA was used to determine if the data were normally distributed. If data were not normally distributed, they were analyzed by the Mann–Whitney *U* test or Kruskal–Wallis *H* test (SPSS 16; Chicago, IL). On the flow rate versus pressure data, a linear regression was carried out to calculate the conventional outflow facility. A nonparametric test for independent samples was used to examine conventional outflow data. In all cases, differences were considered significant at *p* < 0.05.

## 3. Results

### 3.1. The Anterior Segment of Abca1^−/−^ Mice

**Figure 1:**
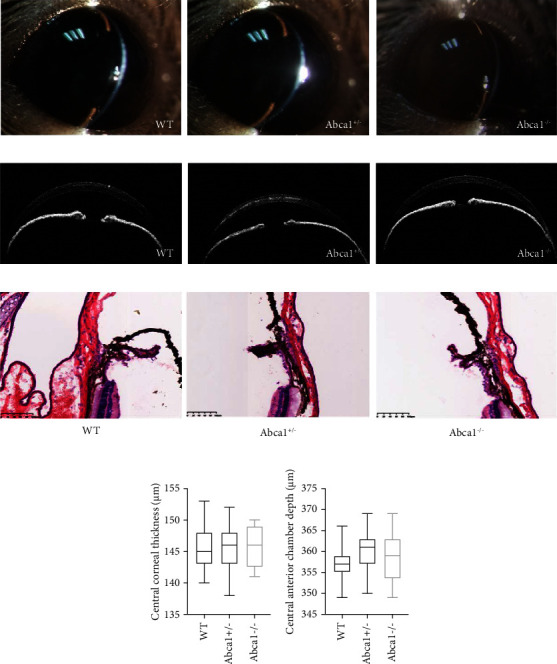
The anterior segments of Abca1^−/−^ mice. (a) Representative images of anterior segments from WT, Abca1^+/−^, and Abca1^−/−^ mice by the slit-lamp microscope. (b) Representative images of anterior segments from WT, Abca1^+/−^, and Abca1^−/−^ mice by the AS-OCT. (c) Representative images of hematoxylin and eosin staining of sections of the outflow tissue. (d) There is no significant difference in the central corneal thickness among WT, Abca1^+/−^, and Abca1^−/−^ mice (*p* > 0.05). (e) There is no significant difference in the central anterior chamber depth among WT, Abca1^+/−^, and Abca1^−/−^ mice (*p* > 0.05). The data were presented by the box-whiskers plot (min to max). (WT, *n* = 15; Abca1^+/−^, *n* = 15; Abca1^−/−^, *n* = 9). WT, wild type.

The cornea of Abca1^−/−^ mice was transparent without turbidness, and the iris was clear. There was no conjunctival congestion, edema, or secretion. No significant morphological difference was observed using a slit-lamp microscope among the Abca1^−/−^, Abca1^+/−^, and WT mice (Figure 1(a)). The anterior chamber angles of Abca1^−/−^ mice were open. Under the hematoxylin and eosin staining of sections of the outflow tissue, there was no significant difference in histology among the Abca1^−/−^, Abca1^+/−^, and WT mice (Figure 1(c)). Furthermore, no significant change in the morphology of anterior segment was found under AS-OCT (Figure 1(b)). After measuring the central corneal thickness and the central anterior chamber depth from the images recorded, the central corneal thickness was 145.8 ± 3.4 *μ*m in Abca1^−/−^ mice, 145.8 ± 3.6 *μ*m in Abca1^+/−^ mice, and 145.6 ± 3.8 *μ*m in WT mice, and the central anterior chamber depths were 359.1 ± 6.5 *μ*m in Abca1^−/−^ mice, 360.1 ± 4.9 *μ*m in Abca1^+/−^ mice, and 356.5 ± 4.6 *μ*m in WT mice, which showed no significant difference among the Abca1^−/−^, Abca1^+/−^, and WT mice (*p* < 0.05, Figures 1(d) and 1(e)).

### 3.2. The IOP of Abca1^−/−^ Mice

**Figure 2:**
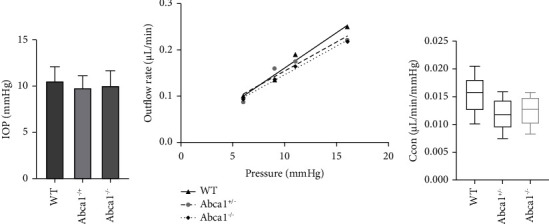
Effect on IOP and conventional outflow. (a) There is no significant difference in IOPs among WT, Abca1^+/−^, and Abca1^−/−^ mice (*p* > 0.05). Error bar means SD (WT, *n* = 15; Abca1^+/−^, *n* = 15; Abca1^−/−^, *n* = 9). (b) The outflow rate of Abca1^−/−^ eyes did not significantly change compared to WT or Abca1^+/−^ at 6, 9, 11, and 16 pressure (*p* > 0.05). (WT, *n* = 7; Abca1^+/−^, *n* = 7; Abca1^−/−^, *n* = 5). (c) There was no significant difference in conventional outflow facility among WT, Abca1^+/−^, and Abca1^−/−^ mice (*p* > 0.05). The data were presented by the box-whiskers plot (min to max) (WT, *n* = 7; Abca1^+/−^, *n* = 7; Abca1^−/−^, *n* = 5). WT, wild type. SD, standard deviation.

Our previous research showed after anterior chamber injection of the ABCA1 agonist GW3965 in mice, IOP could be lowered [14]. In this study, we tried to investigate the IOP of Abca1^−/−^ mice. The average IOP was 10.1 ± 1.6 mmHg of Abca1^−/−^ mice (*n* = 9), 9.8 ± 1.3 mmHg of Abca1^+/−^ mice (*n* = 15), and 10.5 ± 1.6 mmHg of WT mice (*n* = 15), respectively. There was no significant difference in IOP among Abca1^−/−^, Abca1^+/−^, and WT mice (*p* < 0.05, Figure 2(a)).

### 3.3. The Outflow Facility of Abca1^−/−^ Mice

We further examined the impact of ABCA1 deletion on the aqueous humor outflow profile. In Abca1^−/−^ eyes, the outflow rate was 0.093 ± 0.019, 0.136 ± 0.040, 0.164 ± 0.032, and 0.218 ± 0.037 *μ*L per minute at 6, 9, 11, and 16 mmHg, respectively (*n* = 5, Figure 2(b)). In Abca1^+/−^ eyes, the outflow rate was 0.086 ± 0.029, 0.160 ± 0.050, 0.175 ± 0.020, and 0.221 ± 0.030 *μ*L per minute at 6, 9, 11, and 16 mmHg, respectively (*n* = 7, Figure 2(b)). In WT eyes, the outflow rate was 0.100 ± 0.035, 0.135 ± 0.045, 0.190 ± 0.052, and 0.250 ± 0.061 *μ*L per minute at 6, 9, 11, and 16 mmHg, respectively (*n* = 7, Figure 2(b)). The conventional outflow facility for the Abca1^−/−^, Abca1^+/−^, and WT mice were 0.0124 ± 0.0029 *μ*L per min/mmHg, 0.0117 ± 0.0029 *μ*L per min/mmHg, and 0.0154 ± 0.0037 *μ*L per min/mmHg, respectively. There was no significant difference in conventional outflow facility among Abca1^−/−^, Abca1^+/−^, and WT eyes (*p* < 0.05, Figure 2(c)).

### 3.4. Expressions of ABCA1 and Related Protein

**Figure 3:**
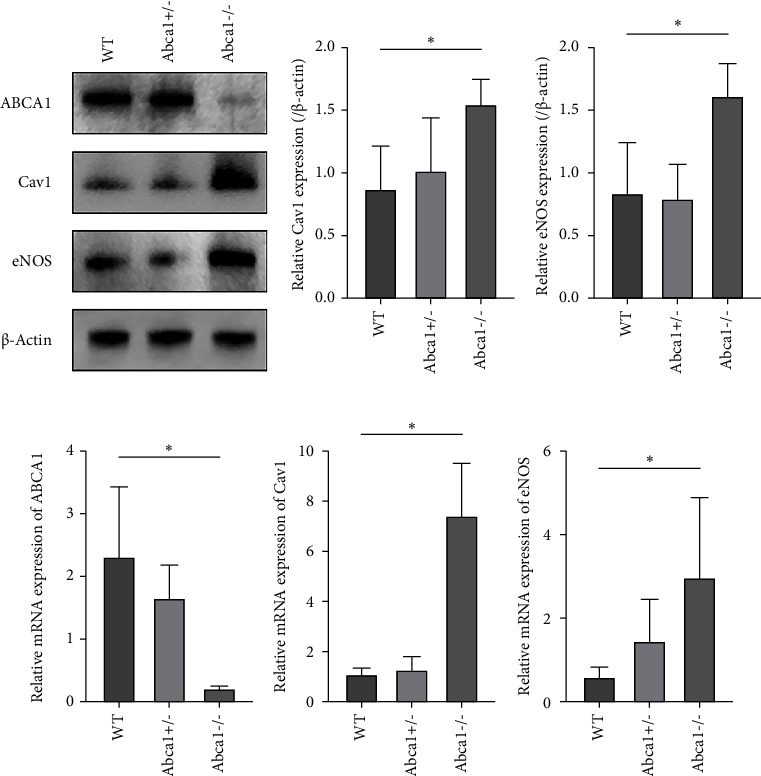
ABCA1 and related protein expression in the outflow tissue. ABCA1 expression in Abca1^−/−^ mice was much lower than that in WT and Abca1^+/−^ mice (a) and (d). RT-qPCR and densitometry analysis of WB results showed increased expressions of Cav1 (b) and (e) and eNOS (c) and (f) in the outflow tissue of Abca1^−/−^ eyes compared to WT and Abca1^+/−^ eyes (WT, *n* = 3; Abca1^+/−^, *n* = 3; Abca1^−/−^, *n* = 3). Results are presented as mean ± SD. ^*∗*^*p* < 0.05. WT, wild type. SD, standard deviation.

**Figure 4:**
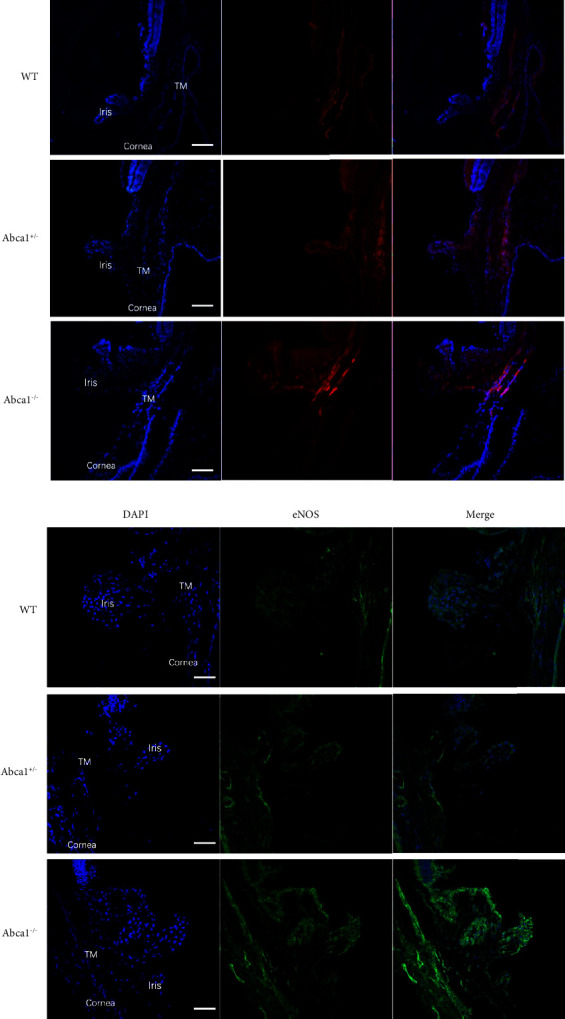
Representative images of immunofluorescence staining of the outflow tissue. (a) Cav1 expression in the outflow tissue. (b) eNOS expression in the outflow tissue. Scale bars, 100 mm. WT, wild type. TM, trabecular meshwork.

We investigated ABCA1 and related protein expressions in the outflow tissue of Abca1^−/−^, Abca1^+/−^, and WT eyes. As expected, RT-qPCR and western blot analysis showed mRNA and protein expressions of ABCA1 in Abca1^−/−^ eyes were significantly lower than those in WT eyes (Figures 3(a) and 3(d)). Then we measured the downstream-related protein expressions as the previous study published [14]. The mRNA expressions of endothelial NO synthase (eNOS) and caveolin-1 (Cav1) were both significantly upregulated in the outflow tissue of Abca1^−/−^ eyes compared to WT eyes by RT-qPCR analysis (*p* < 0.05, Figures 3(e) and 3(f)). Furthermore, the protein expressions of eNOS and Cav1 were also significantly increased in the outflow tissue of Abca1^−/−^ compared to WT eyes (*p* < 0.05, Figures 3(a), 3(b), and 3(c)). The mRNA and protein expressions of ABCA1, Cav1, and eNOS in the outflow tissue of Abca1^+/−^ eyes were not significantly changed comparing with WT eyes (*p* < 0.05, Figures 3(a), 3(b), 3(c), 3(d), 3(e), and 3(f)). To further confirm the protein expressions in the conventional outflow tissue, immunofluorescence staining showed Cav1 (Figure 4(a)) and eNOS (Figure 4(b)) expressions were increased compared with WT eyes. These results showed the Cav1/eNOS pathway was modulated in Abca1^−/−^ mice.

## 4. Discussion

Our study examined the anterior segment in morphology and aqueous humor outflow function in Abca1^−/−^ eyes. The morphology and aqueous humor outflow did not significantly change in Abca1^−/−^ eyes compared to WT eyes. Next, we tried to investigate how ABCA1 was involved in IOP regulation and aqueous humor outflow in the mouse model. Our previous study [14] has found that ABCA1 upregulation in angular aqueous plexus (AAP) cells can decrease Cav1 expression and increase eNOS expression. Conversely, ABCA1 downregulation can increase Cav1 expression and decrease eNOS expression. According to the RT-qPCR, WB, and IF results of our study, Abca1^−/−^ mice lacked the expression of ABCA1 and the expression of Cav1 in outflow tissue was upregulated compared to WT mice. However, the expression of eNOS was also upregulated in Abca1^−/−^ mice, which was different from the above-mentioned result [14].

The morphology, IOP, and aqueous humor outflow of Abca1^−/−^ mice did not show significant change compared with WT mice. This is the first report on the morphology of the anterior segment of Abca1^−/−^ mice. Despite the outflow rate can be increased and the IOP can be reduced by anterior chamber injection of ABCA1 agonist GW3965 in WT mice [14], Abca1^−/−^ eyes did not show the opposite change in IOP and the outflow rate. It indicates that ABCA1 agonist and ABCA1 gene knockout may have different effects on IOP regulation.

Our study showed that ABCA1 deletion increased Cav1 expression. ABCA1 and Cav1 proteins colocalize in cellular membranes in mouse peritoneal macrophages and Cav1 participates in the distribution of ABCA1 within the plasma membrane [18]. By interacting with ABCA1, Cav1 promotes ABCA1 internalization and degradation [19]. In aortic endothelial cells, ABCA1 expression is regulated by Cav1. Suppression of Cav1 decreased ABCA1 expression and reduced cholesterol efflux [20]. ABCA1 overexpression stabilizes Cav1 in colorectal cancer, leading to increased invasive capacities [21]. All the data above indicated ABCA1 and CAV1 can be regulated by each other; however, their underlying mechanism is still unclear.

The regulation of eNOS is complex. A direct protein-protein interaction between eNOS and Cav1 causes a decrease in NO release [22, 23]. Studies using a reconstituted cell culture reveal that eNOS and Cav1 co-transfected cells emit less NO than eNOS alone [24]. Cav1 residues 82–101 carry the functional inhibitory impact on eNOS, whereas the F92 residue is in charge of eNOS inhibition [22, 23]. In endothelial cells, Cav1 siRNA can reduce eNOS protein expression in association with increasing eNOS phosphorylation and nitrate production, which is reversed in cells overexpressing Cav1. So Cav1 maintains eNOS expression and regulates its activity [25]. Also, it has been reported that eNOS knockout mice have elevated IOP and their conventional drainage is significantly lower [26], which is consistent with human evidence revealing glaucoma-related polymorphisms in the eNOS gene [27–29]. And Cav1 deficiency results in increased eNOS activity, which means the control of IOP requires the interplay of eNOS and Cav1 [30, 31].

In our study, we found ABCA1 deletion increased Cav1 expression, which should decrease eNOS expression based on the research stated above, but we saw an increase in eNOS in our ABCA1 knockout mouse model. The reason for this discrepancy might be the increased Cav1 is not fully functional and the dysfunction of Cav1 leads to the increase in eNOS. So the outflow rate of aqueous humor did not decrease and IOP was not elevated in Abca1^−/−^ mice with the increased eNOS activity.

Compared with previous research [14], we edited the genome for ABCA1 gene knockout instead of RNA interference(RNAi)-mediated knockdown using short hairpin RNA (shRNA). ABCA1 is one of the several proteins involved in cholesterol homeostasis [5]. Numerous organs may be impacted by ABCA1 deletion, which also plays a role in the pathophysiology of a wide range of disorders. So eliminating ABCA1 gene function might cause changes in homeostasis. Moreover, In Vivo models seemed to be a more complex system than In Vitro studies and functional protein knockout in the whole body caused different results compared to using the ABCA1 agonist or lentiviral ABCA1-shRNA in the cell studies. The use of In Vivo models enables the investigation of whole-organism complexity because the visual system and other body systems (immune function, nervous system, etc.) are intact [32]. Glaucoma is a complex systemic disease so physiological responses should involve many cell types and extracellular effects [2]. Now it is still not clear how ABCA1 affects aqueous humor outflow. At present, there are few studies on the function and mechanism of ABCA1 in glaucoma. Understanding the physiological and pathological involvement of ABCA1 in glaucoma and the potential benefits of targeting ABCA1 for the treatment of glaucoma still require more research.

In our study, we employed chloral hydrate as the sole sedative in two procedures: slit-lamp microscopic examination and AS-OCT scanning, because both procedures were noncontact and noninvasive in nature. Intraperitoneal administration of chloral hydrate could result in adverse effects such as adynamic ileus, gastric ulcers, and peritonitis in laboratory animals [33]. We observed no systemic adverse effects following intraperitoneal administration of chloral hydrate in our research. Additionally, no abnormal signs were noted upon awakening from the induced state of hypnosis.

## 5. Conclusion

ABCA1 deletion does not affect IOP and aqueous humor outflow function but the Cav1/eNOS/NO pathway is changed in Abca1^−/−^ mice.

## Data Availability

The data that support the findings of this study are available on request from the corresponding author.
